# Baseline Xpert MTB/RIF ct values predict sputum conversion during the intensive phase of anti-TB treatment in HIV infected patients in Kampala, Uganda: a retrospective study

**DOI:** 10.1186/s12879-021-06220-6

**Published:** 2021-06-01

**Authors:** Juliet Namugenyi, Joseph Musaazi, Achilles Katamba, Joan Kalyango, Emmanuel Sendaula, Andrew Kambugu, Jan Fehr, Barbara Castelnouvo, Yukari C. Manabe, Willy Ssengooba, Christine Sekaggya-Wiltshire

**Affiliations:** 1 Infectious Diseases Institute, College of Health Sciences, Makerere University, Kampala, Uganda; 2 Clinical Epidemiology Unit, School of Medicine, College of Health Sciences, Makerere University, Kampala, Uganda; 3 Department of Pharmacy, College of Health Sciences, Makerere University, Kampala, Uganda; 4 Division of Infectious Diseases and Hospital Epidemiology, University Hospital Zurich, University of Zurich, Zurich, Switzerland; 5 Division of Infectious Diseases, Department of Medicine, Johns Hopkins University School of Medicine, Baltimore, MD USA; 6 Department of Medical Microbiology, College of Health Sciences, Makerere University, Kampala, Uganda; 7 Makerere Lung Institutes, College of Health Sciences, Makerere University, Kampala, Uganda

**Keywords:** *Mycobacterium tuberculosis*, HIV, Xpert MTB/RIF, Baseline ct values, Time to positivity, Colony count

## Abstract

**Background:**

In resource-limited settings, sputum smear conversion is used to document treatment response. Many People living with HIV (PLHIV) are smear-negative at baseline. The Xpert MTB/RIF test can indirectly measure bacterial load through cycle threshold (ct) values. This study aimed to determine if baseline Xpert MTB/RIF could predict time to culture negativity in PLHIV with newly diagnosed TB.

**Methods:**

A subset of 138 PLHIV from the ‘SOUTH’ study on outcomes related to TB and antiretroviral drug concentrations were included. Bacterial load was estimated by Mycobacterium Growth Indicator Tubes (MGIT) culture time-to-positivity (TTP) and Lowenstein Jensen (LJ) colony counts. Changes in TTP and colony counts were analyzed with Poisson Generalised Estimating Equations (GEE) and multilevel ordered logistic regression models, respectively, while time to culture negativity analysed with Cox proportional hazard models. ROC curves were used to explore the accuracy of the ct value in predicting culture negativity.

**Results:**

A total of 81 patients (58.7%) were males, median age 34 (IQR 29  – 40) years, median CD4 cell count of 180 (IQR 68  – 345) cells/μL and 77.5% were ART naive. The median baseline ct value was 25.1 (IQR 21.0  – 30.1). A unit Increase in the ct value was associated with a 5% (IRR = 1.05 95% CI 1.04  – 1.06) and 3% (IRR = 1.03 95% CI 1.03  – 1.04) increase in TTP at week 2 and 4 respectively. With LJ culture, a patient’s colony grade was reduced by 0.86 times (0R = 0.86 95% CI 0.74  – 0.97) at week 2 and 0.84 times (OR = 0.84 95% CI 0.79  – 0.95 *P* = 0.002) at week 4 for every unit increase in the baseline ct value. There was a 3% higher likelihood of earlier conversion to negativity for every unit increase in the ct value. A ct cut point ≥28 best predicted culture negativity at week 4 with a sensitivity of 91. 7% & specificity 53.7% while a cut point ≥23 best predicted culture negativity at week 8.

**Conclusion:**

Baseline Xpert MTB/RIF ct values predict sputum conversion in PLHIV on anti-TB treatment. Surrogate biomarkers for sputum conversion in PLHIV are still a research priority.

## Background

In 2019, it is estimated that ten million people fell ill with tuberculosis (TB) worldwide and 1.4 million deaths occurred, 8.2% of these were people living with HIV (PLHIV) [1]. In 2018, PLHIV constituted 40% of overall country TB incidence in Uganda [2]. Despite an improvement in the treatment coverage, TB transmission is still high. Early identification of TB cases likely to have slow treatment response, who experience treatment failure or relapse would enable clinicians to implement individualized treatment plans in order to achieve higher cure rates, reduce on transmission and achieve WHO targets. The initial sputum bacterial load estimated by smear or culture provides a measure of bacterial burden at treatment initiation and is a fairly good indicator of time to sputum conversion [3–5]. However, HIV co-infected participants are often smear-negative at baseline [6, 7] and rarely have cultures obtained due to expense, need for specialized labs and long turnaround time [8]. The Xpert MTB/RIF test (Cepheid, Sunnyvale, CA) is widely used in TB diagnosis [9] due to its improved sensitivity over smear microscopy and a short turnaround time [6, 7, 10, 11]. It also provides a measure of bacterial load through cycle threshold (ct) values [12]. Ct values demonstrate the number of PCR cycles that the MTB DNA goes through to reach the level of detection; higher ct values correlate with lower bacterial loads [13]. Ct values have been demonstrated to have strong correlation with bacterial loads measured by smear or culture [14]. Shenai et al. in South Africa [5] demonstrated that baseline ct values provided a good estimate of time to sputum culture conversion and likelihood of relapse in an HIV-negative population. HIV infection is a risk factor for poor treatment response especially in those with low CD4 cell counts [15–18]. This study sought to determine if baseline ct values predict time to sputum culture conversion and correlate with decreasing bacterial load measured by Mycobacteria Growth Indicator Tube (MGIT) time to positivity (TTP) and Löwenstein–Jensen (LJ) colony counts during the first two months of treatment in HIV infected participants with severe immunosuppression. PLHIV frequently have paucibacillary TB and varying levels of immunity which may affect rate of bacterial clearance. Distinguishing between high and low bacterial load in relation to this population is paramount in effective monitoring and prediction of bacteriological and clinical TB response. Therefore, we also explored the sensitivities and specificities of various ct cut offs to confirm how correct the predictions of negativity are at different time points of treatment.

## Methods

### Study design and setting

This was a subset of a cohort study conducted at the TB-HIV integrated clinic of the Infectious Diseases Institute (IDI), Kampala, Uganda [19] between January 2013 and May 2015, called ‘Study on Outcomes related to TB and HIV drug Concentrations in Uganda’ (SOUTH) (Sekaggya-Wiltshire et al., 2017). Participants with a first episode of pulmonary TB were included in the SOUTH study and excluded if they had drug resistant TB, TB requiring treatment for more than 6 months for example tuberculous meningitis and spinal TB, mycobacteria other than TB, renal or liver failure or if they were pregnant. For this analysis, we included all participants who had a baseline Xpert MTB/RIF test and more than three subsequent follow-up sputum culture results within the first two months of treatment. Participants that had invalid Xpert MTB/RIF results or contaminated sputum cultures were excluded.

### Laboratory methodology

Xpert testing, sputum smear and cultures were performed on all participants. Laboratory testing was performed at the College of American Pathologists (CAP-)-accredited Makerere University Tuberculosis Laboratory. Sputum specimens were decontaminated with sodium hydroxide N-acetyl-L-cysteine (NAOH/NALC). At baseline, the decontaminated sputum was split into two equal aliquots for Xpert testing and culture. For the Xpert MTB/Rif test, 1 ml of sputum was treated with 2 ml of sample reagent and processed according to manufacturer’s Standard Operating Procedures (SOPs) (Cepheid USA). The Xpert MTB/RIF ct values were extracted from the GeneXpert software database. A mean ct value for each participant was calculated from the ct readouts of the five *rpoB* gene probes.

For culture, the specimens were concentrated by centrifuging at 3000 rpm for 15 min, supernatant discarded and the pellet re-suspended in sterile phosphate buffer to 2 ml [20]. We inoculated 0.5 ml of the remaining suspension into BACTEC MGIT 960 culture tube and 0.5 ml on LJ slants. Following inoculation, the MGIT culture tubes were incubated at 37 °C in the BACTEC MGIT 960 instrument according to the laboratory standard operating procedure and monitored daily until growth was detected or for a maximum of 42 days in case of negative cultures. Time-to-positivity (TTP) was then recorded as the number of days between inoculation of specimen into culture tube and detection of growth. Specimens inoculated on LJ slants were incubated at 37 °C, checked weekly for growth or for 8 weeks for negative cultures and the bacterial colonies were then counted. Sputum cultures were performed at 2, 8 and 24 weeks following TB treatment initiation. Due to availability of more funds, participants enrolled in the latter part of the study received more intensive microbiological follow-up with additional sputum cultures at 4,6,10 and 12 weeks.

### Statistical analysis

The baseline characteristics of the participants were described using proportions for categorical variables and median and inter-quartile ranges for continuous data. The primary outcomes were; 1) change in MGIT TTP and LJ colony counts during the first two months of TB treatment and 2) time in weeks to culture negativity (time between initiation of TB treatment and sputum culture conversion). For MGIT TTP, an imputed value of 45 (value above highest TTP recorded in this cohort) was assigned to any negative cultures and the changes in TTP analysed with Poisson Generalised Estimating Equations (GEE) models (with the ‘log’ link). LJ colony count data was graded according to current WHO grading criteria: > 200 (4+), 100  – 200 (3+), 10  – 100 (2+), < 10 (1+) and multilevel mixed effects ordered logistic regression used to analyse the changes in colony count grades. Cox proportional hazard regression models with right censoring were used to analyze the time to culture negativity with survival time measured as the time from initiation on anti-TB treatment until participant was censored or got first culture negative result with no subsequent positive cultures. Participants were censored for loss to follow up or death. Receiver operating characteristic (ROC) curve analysis was used to explore the accuracy of baseline ct values in predicting sputum culture status at weeks 4, 8 and 24. All analysis was done with Stata V13.0 [21].

## Results

### Baseline characteristics of the study population

**Fig. 1:**
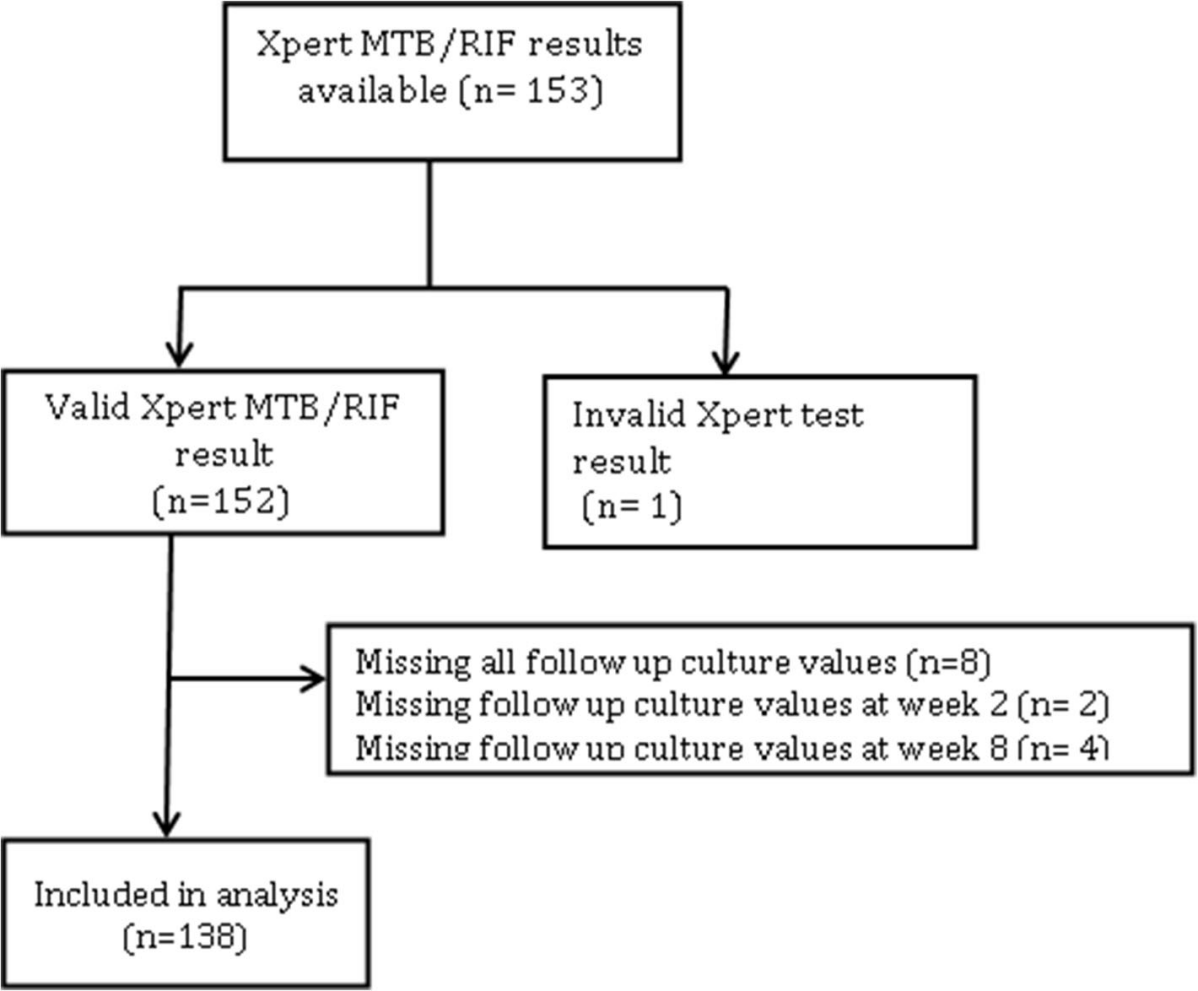
Flow chart showing participant inclusion and exclusion process

Of the 268 SOUTH study participants, 153 participants had an Xpert positive pulmonary TB diagnosis. We excluded 1 participant with invalid Xpert MTB/RIF results and those with contaminated cultures (8 had contaminated MGIT cultures at all 5 time points during the first 8 weeks, 6 had contaminated cultures at either or both week 2 and 8) (Fig. 1). Data from the remaining 138 participants was analyzed.

Of the 138 participants analyzed, 81 (58.7%) participants were males with a median age of 34 (IQR 29  –  40) years, median CD4 cell count of 180 (IQR 68  – 345) cells/μl, and 107 (77.5%) were ART-naive at baseline. Fourteen (11.8%) were smokers and 124 (93.24%) had abnormal chest X-ray findings with 30 (21.7%) having cavities. The overall median baseline Xpert MTB/RIF ct value was 25.1 (IQR: 20.9  – 30.3), but participants with cavitary disease had a lower median baseline Xpert ct value 22. 4 (IQR: 19.0  – 23.9, *P* = 0.0007) and a higher median CD4 cell count 269 cells/μL (IQR: 203  – 397, *P* = 0.0049) compared to those with no cavities (Table 1). In this cohort, majority 87 (63%) of the participants were smear negative. Smear negative participants had a higher median TTP 45 (IQR: 14  –  42) and a higher median ct value 21.4 (IQR: 18.3  – 27.7) compared to smear positive participants with 10 (IQR: 7  – 18) and 27.2 (IQR: 22.9  – 31.1) respectively. Likewise, majority 70(80.5%) of smear negative participants had between 1+ and 3+ colony count grades on LJ while majority 27(52.9%) of smear positive participants had 4+ grades (Table 2).

**Table 1:** Sociodemographic and clinical baseline characteristics of the study population

Baseline characteristics	Participants (***N*** = 138)
Male sex, N (%)	81 (58.7)
On ART at baseline, N (%)	31 (22.5)
Smokers^a^, N (%)	14 (11.8)
BMI, median (IQR), kg/m^2^	19.3 (17.7 – 21.7)
BMI > 18.5 kg/m^2^	92 (66.7)
Abnormal Chest X-ray^a^ N (%)	124 (93.2)
Cavitation, N (%)^a^	30 (21.7)
Age median (IQR), years	34 (29 – 40)
CD cell count, median (IQR), cells/μL	192 (69 – 361)
Xpert MTB/RIF ct value, median (IQR)	25.1 (21.0 – 30.1)

*Abbreviations*: *ART* antiretroviral therapy, *BMI* body mass index, *IQR* interquartile range

^a^Missing data: history of smoking (19/138), chest x-ray result (5/138)

**Table 2:** Smear microscopy profile of participants in relation to Xpert MTB/RIF CT value, MGIT TTP and LJ colony grades at baseline

Category	Smear grade	Frequency n (%) N = 138	Baseline CT value Mean ± sd	Baseline TTP Mean ± sd, days	LJ Colony grade				
					Neg (n)	1+ (n)	2+ (n)	3+ (n)	4+ (n)
Smear positive^a^	Scanty	4 (6.3)	25.7 ± 6.6	19 ± 16	1	5	0	0	0
	1+	12 (8.7)	24.7 ± 6.9	13 ± 10	1	5	3	2	1
	2+	8 (5.8)	23.9 ± 5.4	7 ± 2	0	0	1	3	4
	3+	24 (17.4)	21.6 ± 5.9	5 ± 3	0	0	1	1	22
Smear Negative	87 (58.9)		26.9 ± 5.0	22 ± 16	4	33	27	16	7

^a^ Missing data: Smear positive (1/51- missing smear grade)

### Association between baseline Xpert MTB/RIF ct value and changes in MGIT TTP and colony count during the first two months of treatment

**Fig. 2:**
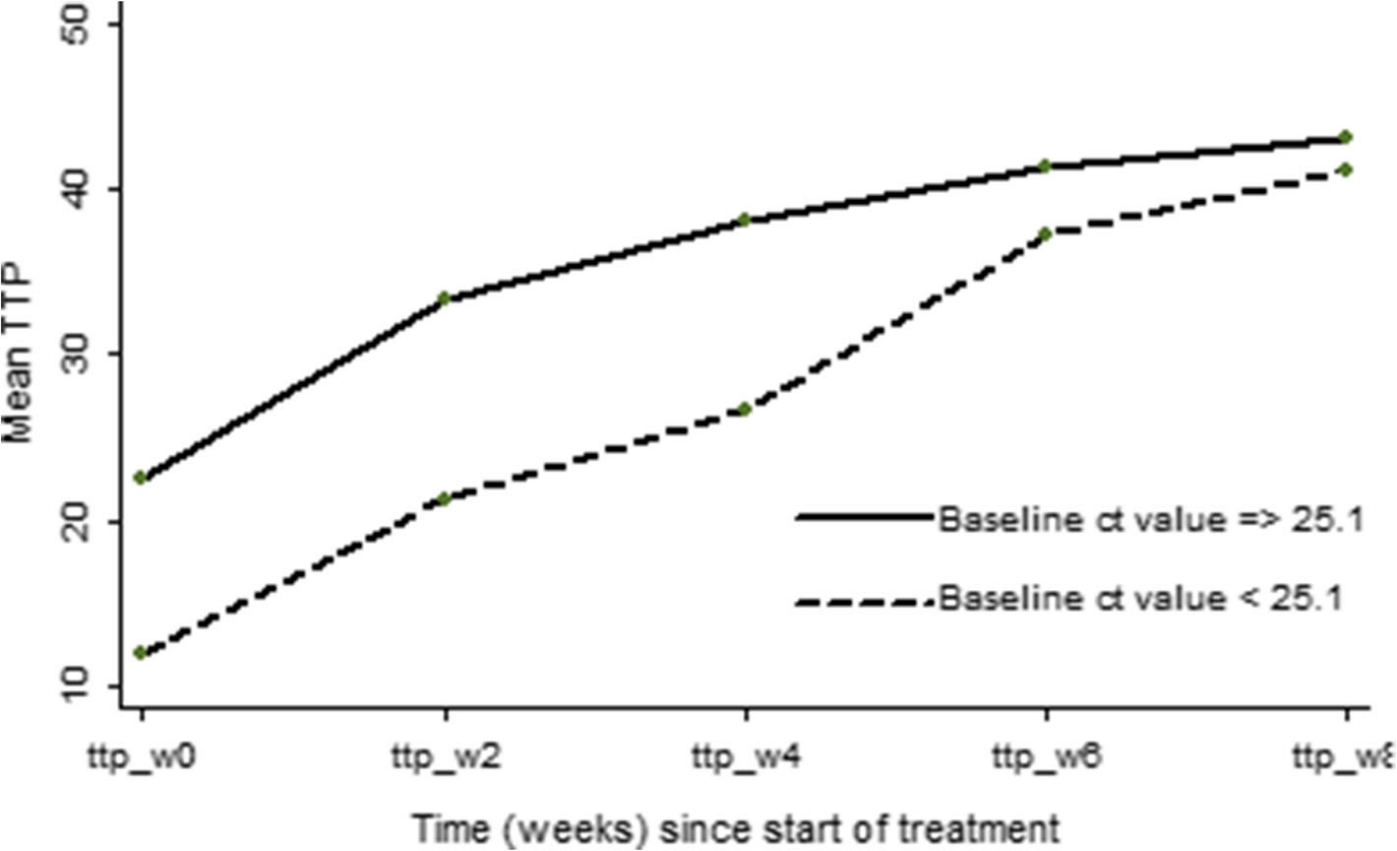
TTP during the first two months of treatment categorized by the median ct value

Participants with a baseline ct value < 25.1 had lower TTP at baseline, week 2, 4 and 6 following initiation of TB treatment compared to those > 25.1 (Fig. 2). After adjusting for the CD4 cell count, cavitation, ART status, BMI, smoking history, age and sex, a higher baseline ct value was associated with having higher TTP at baseline, week 2 (IRR:1.05, 95% CI 1.04  – 1.06, *P* <  0.001) and week 4 (IRR:1.03, 95% CI 1.03  – 1.04, *P* <  0.001) (Table 3). Similarly, a higher baseline ct value was also associated with having a reduced colony count (lower bacterial load) at baseline until the 4th week following treatment initiation (OR: 0.86, 95% CI 0.74  – 0.97, *P* = 0.002 at week 2) and (OR: 0.84, 95% CI 0.79  – 0.95, *P* = 0.017 at week 4) (Table 4). This association was lost after the 4th week of treatment. Having cavities was associated with a shorter TTP and higher colony count at baseline (Tables 3 and 4), while having a baseline CD4 cell count ≥200 cells/μl was associated with having a longer TTP at baseline (Table 3).

**Table 3:** Factors associated with TTP during the first two months of treatment

Variable	Unadjusted		Adjusted	
	IRR (95% CI)	***P***-value	IRR (95% CI)	P-value
**Baseline CT value**^a^				
Week 0	1.02 (1.02 – 1.03)	**< 0.001**	1.05 (1.04 – 1.06)	< 0.001
Week 2	1.04 (1.03 – 1.04)	< 0.001	1.03 (1.02 – 1.04)	**< 0.001**
Week 4	1.03 (1.02 – 1.03)	< 0.001	1.02 (1.01 – 1.03)	**< 0.001**
Week 6	1.01 (1.00 – 1.02)	0.009	1.00 (0.99 – 1.01)	0.376
Week 8	1.01 (1.00 – 1.01)	0.001	1.00 (0.99 – 1.01)	0.296
**CD4 cell count**				
≤ 200 cells/μL	**Ref**			
> 200 cells/μL	1.10 (1.06 – 1.14)	< 0.001	1.05 (1.00 – 1.09)	** 0.034**
**Cavitation**				
No	**Ref**			
Yes	0.75 (0.72–0.79)	< 0.001	0.83 (0.79 – 0.88)	**< 0.001**
**ART status**				
On ART	**Ref**			
Not on ART	1.07 (1.03 – 1.12)	0.001	1.03 (1.00 – 1.06)	0.098
**BMI**				
≤ 18.5	**Ref**			
> 18.5	0.93 (0.89 – 0.97)	< 0.001	0.72 (0.56 – 1.10)	0.067
**Sex**				
Male	**Ref**			
Female	0.99 (0.95 – 1.03)	0.596		
**Age**	1.00 (0.99 – 1.00)	0.444		

^a^Effect of baseline CT value on change in TTP at weeks 2, 4, 6 and 8 with reference to the baseline TTP

**Table 4:** Factors associated with LJ colony count during the first two months of treatment

Variable	Unadjusted		Adjusted	
	OR (95% CI)	P-value	OR (95% CI)	P-value
**Baseline ct value**^a^				
Week 0	0.91 (0.88 – 0.94)	< 0.001	0.84 (0.76 – 0.92)	< 0.001
Week 2	0.84 (0.76 – 0.92)	< 0.001	0.86 (0.79 – 0.95)	** 0.002**
Week 4	0.83 (0.72 – 0.95)	0.007	0.86 (0.75 – 0.98)	** 0.026**
Week 6	0.86 (0.73 – 1.00)	0.057	0.89 (0.76 – 1.04)	0.139
Week 8	0.88 (0.73 – 1.06)	0.167	0.91 (0.75 – 1.09)	0.302
**Cavitation**				
No	**Ref**			
Yes	2.54 (1.56 – 4.13)	< 0.001	3.56 (1.33 – 9.54)	** 0.012**
**CD4 cell count**				
≤ 200 cells/μL	**Ref**			
> 200 cells/μL	0.59 (0.38 – 0.92)	0.020	0.86 (0.63 –1.17)	0.073
**ART status**				
On ART	**Ref**			
Not on ART	0.92 (0.54 – 1.57)	0.758		
**Body Mass Index**				
≤ 18.5	**Ref**			
> 18.5	1.15 (0.71 – 1.85)	0.575		
**Sex**				
Male	**Ref**			
Female	0.85 (0.54 – 1.34)	0.484		
**Age**	0.99 (0.96 – 1.02)	0.473		

^a^Effect of baseline CT value on change in colony count at weeks 2, 4, 6 and 8 with reference to the baseline count

### Time to culture negativity analysis

**Fig. 3:**
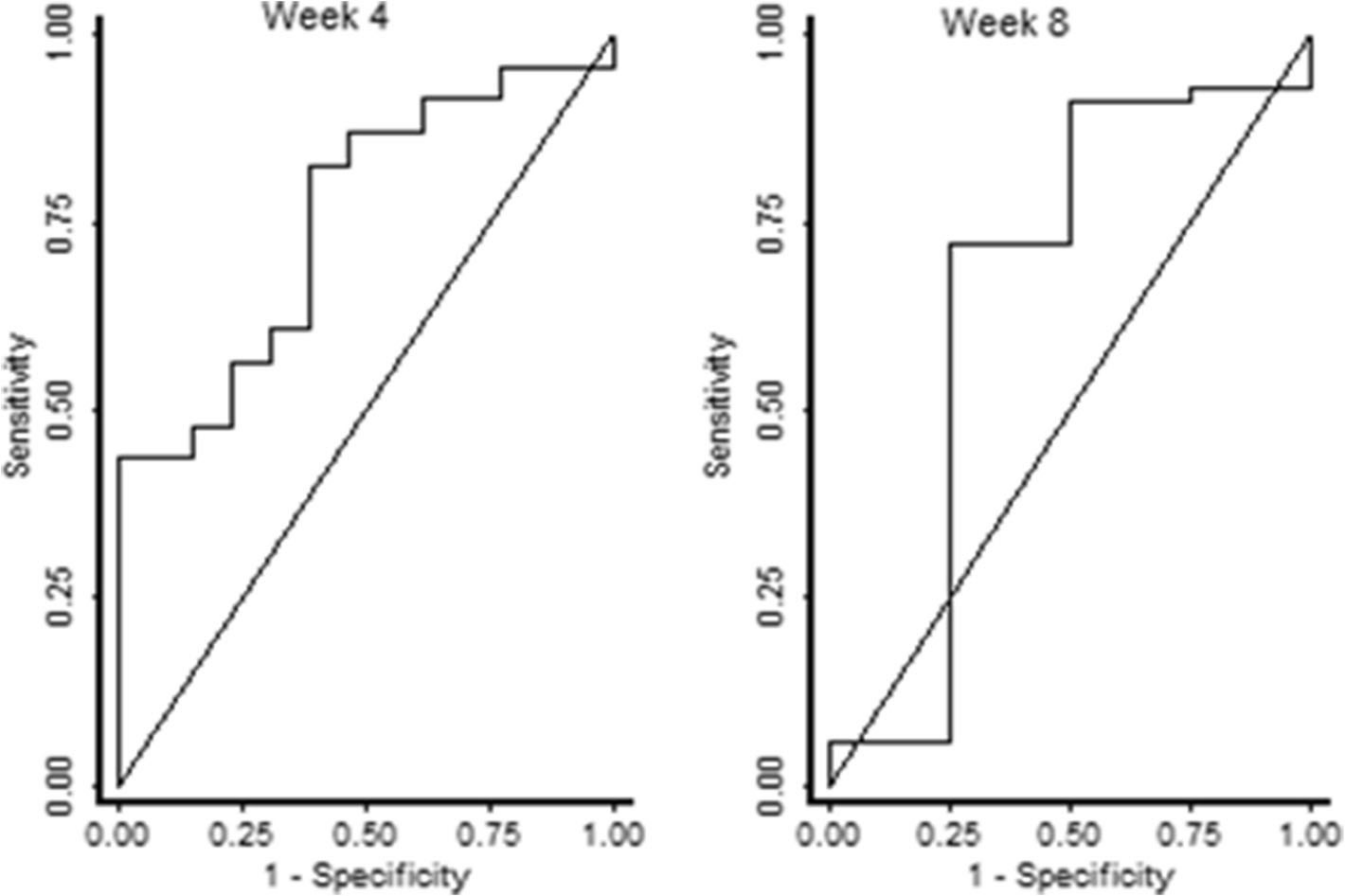
ROC curves for baseline ct value relative to LJ culture negativity

**Fig. 4:**
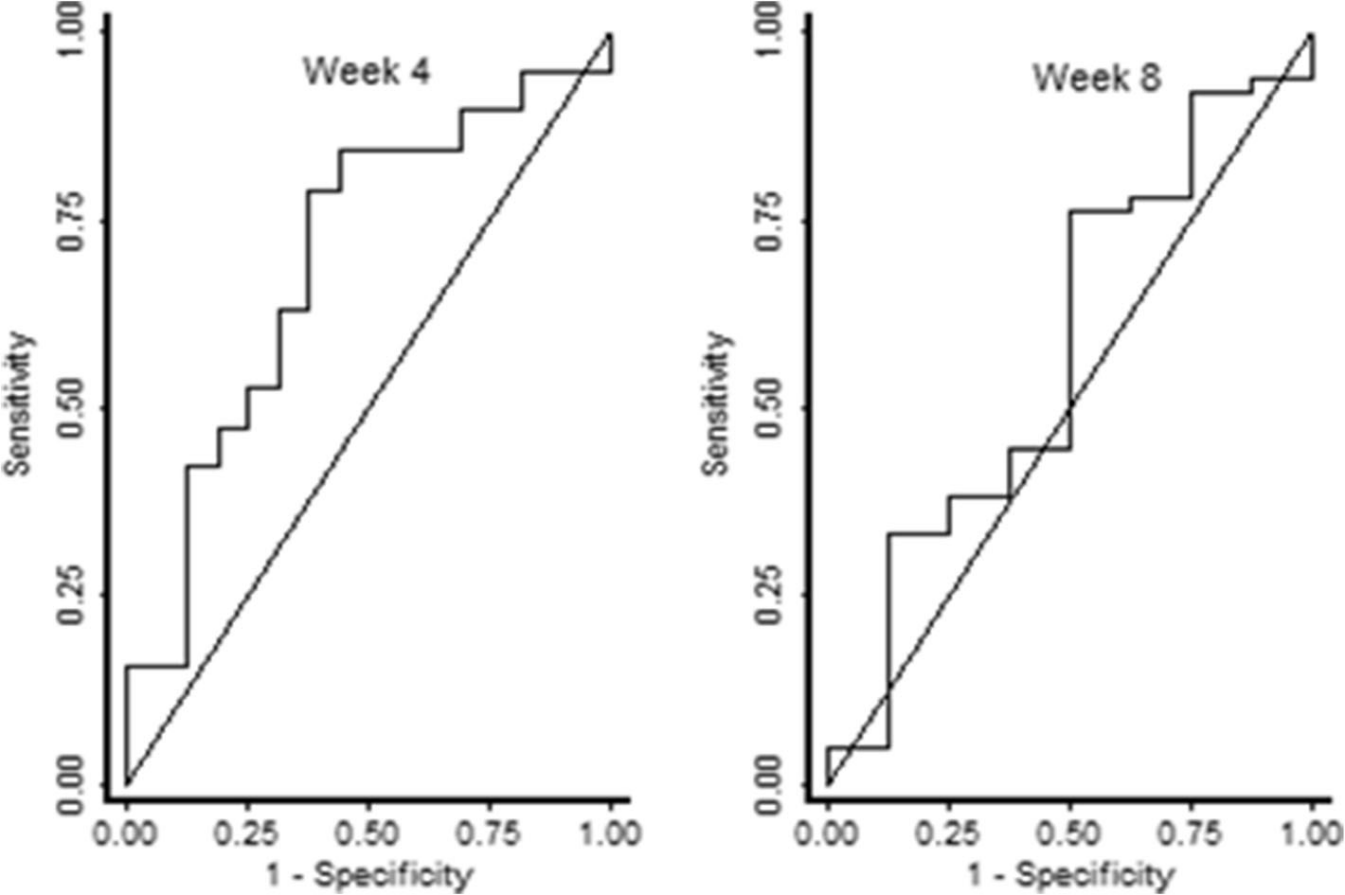
ROC curves for baseline ct value relative to MGIT culture negativity

ROC curve results showed that the baseline ct value most accurately predicted culture status at week 4 with AUC: 77.6, 95% CI 66.0–89.2 and AUC: 71.4, 95% CI 57.12–85.6 for LJ and MGIT culture respectively (Figs. 3 and 4). A ct cut point ≥28 had the highest sum of sensitivity (91.7%) and specificity (53.7%) to predict culture negativity at this point. At week 8, a ct value cut off of 23 had the highest sum of sensitivity (83.3%) and specificity (65.2%) with AUC: O.69, 95% CI 0.41–0.96 for LJ and AUC: O.60, 95% CI 0.42–0.79 for MGIT.

**Fig. 5:**
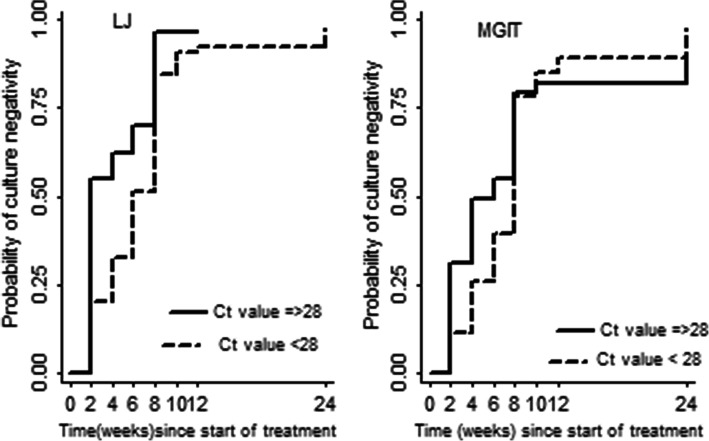
Cumulative probability of conversion to negativity for two groups categorized at baseline ct value 28

Using MGIT, 17% were culture negative by week 2, 78% by week 8 and 95% by week 24 while with LJ culture, 29, 87 and 97% were negative by week 2, 4 and 8 respectively. Participants who had baseline ct value ≤28.0 had a lower probability of conversion to culture negativity during the first two months of treatment compared to those > 28.0 (Fig. 5). A higher baseline ct value was associated with an increased likelihood of earlier conversion to negativity using LJ during the first two months of treatment (HR: 1.03, 95% CI 01.00–1.06, *P* = 0.032) (Table 5) but there was no significant association after week 8.

**Table 5:** Factors associated with time to LJ culture negativity during the first two months of treatment

Variable	Unadjusted		Adjusted	
	HR (95% CI)	P-value	HR (95% CI)	P-value
**Baseline ct value**	1.04 (1.01–1.07)	0.014	1.03 (1.00–1.06)	**0.032**
**CD4 cell count**				
≤ 200	**Ref**			
> 200	1.35 (0.98–1.86)	0.060	1.38 (1.00–1.89)	**0.050**
**ART status**				
On ART	**Ref**			
Not on ART	1.46 (0.98–2.18)	0.064	1.20 (0.79–1.32)	0.068
**Cavitation**				
No	**Ref**			
Yes	0.87 (0.61 – 1.23)	0.430		
**Age**	0.99 (0.97–1.02)	0.703		
**Sex**				
Male	**Ref**			
Female	0.88 (0.64–1.20)	0.416		
**Smoking status**				
Yes	**Ref**			
No	1.11 (0.01–55.32)	0.966		
**Body Mass Index**				
` ≤ 18.5	**Ref**			
> 18.5	0.95 (0.67–1.34)	0.769		

Having a CD4 cell count ≥200 cells/μl at baseline was associated with a higher likelihood of earlier conversion to negativity using LJ during the first two months of treatment compared to having < 200 cells/μl at baseline (*P* = 0.050) (Table 5). Cavitation, ART status, BMI, smoking status, age and sex had no significant association with time to culture negativity.

## Discussion

We found that the baseline Xpert MTB/RIF ct value was significantly associated with; 1) change in bacterial load measured by TTP and colony counts up to 4 weeks following treatment initiation, 2) eventual time to culture negativity in the first two months of treatment. ROC analysis suggested that the baseline ct value most accurately predicted culture conversion at week 4. Our findings concur with a study done in South Africa that reported high baseline ct values to increase the likelihood of earlier culture conversion, with a ct value ≥30 predicting culture negativity (sensitivity 88.2% and specificity 51.4% at week 4 and a sensitivity of 78.9% and specificity 78.9% at week 8) [5]. However, unlike the Shenai study where it was still a good predictor until week 24, the baseline ct value predicted sputum clearance only during the first two months of treatment in this study. This was probably because the Shenai study population was HIV negative with relatively similar immune responses while the current study comprised of HIV-infected participants who may have variable immune reconstitution related to timing of ART initiation, and other factors related to treatment like adherence. In addition, as a cohort of treatment sensitive participants improve on treatment, it is likely that they gradually become clinically similar (cohort effect), although we have not found other studies to indicate this is a possible reason and should be evaluated in future studies. A relatively lower specificity (53.7%) of ct for predicting culture negativity at week 4 was probably because a cut point of 28 was much higher than the overall median ct value (25.1). This needs to be explored further. The association with time to negativity was evident with only LJ culture. Because MGIT culture is demonstrated to have a higher yield, positivity rate and shorter mean times to detection than LJ [22, 23], the Xpert MTB/RIF accuracy in this study could have been more similar to that of LJ than MGIT implying a higher correlation between ct values and colony counts than with TTP. In a previous study conducted among Ugandan presumptive TB patients, a slightly higher correlation (r = − 0.38) was reported between Xpert ct values and LJ culture grades than the correlation between xpert ct values and MGIT TTP (r = 0.37), although general correlation reported in this study between xpert ct and culture was very low [24].

A CD4 cell count ≥200 cells/μl was a significant predictor of high baseline bacterial load and delayed time to culture negativity. This was probably caused by the cavitary TB participants since they had higher CD4 cell count and higher bacterial load (as estimated by the baseline ct value). Pulmonary TB infection in people with an intact immunity triggers a response that leads to cavitation [25, 26] which in turn creates a perfect medium for increasing bacterial growth [27–29].

Our study had some limitations: Differences in sputum quality at any time point could have had effect on ct values, colony count and ttp and impacted results. We also did not have the opportunity to assess the effect of other important confounding factors like drug adherence, the WHO HIV clinical stage and other opportunistic infections during the period of interest that have been shown to have impact on sputum conversion.

## Conclusion

We have demonstrated that initial bacterial load estimated using the baseline Xpert MTB/RIF ct value is a good predictor of time to sputum conversion in the first two months of treatment in TB/HIV infected participants on first line anti-TB treatment. In countries where the Xpert MTB/RIF test is still used as the diagnostic test for TB in HIV-infected participants, the baseline ct values can help identify participants likely to have slower sputum conversion, which may have implications for TB transmission taking into account individual patient health states. Although a ct value > 28 had a high sensitivity for identifying HIV/TB co-infected participants that are likely to be culture negative by week 4, it’s specificity was low. Our study has provided more basis for future studies on the use of the ct values derived from advanced molecular based technologies like the Xpert MTB/RIF ultra. Since the Xpert MTB/RIF Ultra is now more frequently used and has a lower limit of detection with two different multicopy amplification targets (IS*6110* and IS*1081*) instead of the *rpo* region used by Xpert MTB/RIF, further research is needed to explore its ct value relationship with culture conversion. The correlation of ct values with LJ negativity alone warrants further research. Biomarkers to predict sputum conversion during entire 24 weeks of treatment are also still a research priority.

## Data Availability

The Dataset analysed during the current study is available from the corresponding author upon reasonable request.

## Abbreviations

ART: Antiretroviral therapy
CD4: Cluster of Differentiation 4
Ct: Cycle threshold
DNA: Deoxyribonucleic acid
DOTS: Directly Observed Treatment Short course
GEE: Generalized Estimating Equations.
HIV: Human Immunodeficiency Virus
IDI: Infectious Diseases Institute
IRIS: Immune reconstitution Inflammatory Syndrome
LJ: Lowenstein-Jensen
MGIT: Mycobacteria Growth Indicator Tube
MTB: *Mycobacterium tuberculosis*
PCR: Polymerase Chain Reaction
PLHIV: People Living with Human Immunodeficiency Virus
RHZE: Rifampicin, Isoniazid, Pyrazinamide and Ethambutol
SOUTH: Study on outcomes related TB and antiretroviral drug concentrations
TB: Tuberculosis
TTP: Time to positivity
WHO: World Health Organization
Xpert MTB/RIF: GeneXpert *Mycobacterium tuberculosis*/ Rifampicin resistance
